# *Bartonella* type IV secretion effector BepC induces stress fiber formation through activation of GEF-H1

**DOI:** 10.1371/journal.ppat.1009065

**Published:** 2021-01-28

**Authors:** Chunyan Wang, Haoran Zhang, Jiaqi Fu, Meng Wang, Yuhao Cai, Tianyun Ding, Jiezhang Jiang, Jane E. Koehler, Xiaoyun Liu, Congli Yuan

**Affiliations:** 1 School of Agriculture and Biology, Shanghai Jiao Tong University, Shanghai, China; 2 Shanghai Key Laboratory of Veterinary Biotechnology, Shanghai, China; 3 Institute of Analytical Chemistry, College of Chemistry and Molecular Engineering, Peking University, Beijing, China; 4 Department of Medicine, Division of Infectious Diseases, and the Microbial Pathogenesis and Host Defense Program, University of California, San Francisco, California, United States of America; 5 Department of Microbiology, School of Basic Medical Sciences, Peking University Health Science Center, Beijing, China; Purdue University, UNITED STATES

## Abstract

*Bartonella* T4SS effector BepC was reported to mediate internalization of big *Bartonella* aggregates into host cells by modulating F-actin polymerization. After that, BepC was indicated to induce host cell fragmentation, an interesting cell phenotype that is characterized by failure of rear-end retraction during cell migration, and subsequent dragging and fragmentation of cells. Here, we found that expression of BepC resulted in significant stress fiber formation and contractile cell morphology, which depended on combination of the N-terminus FIC (filamentation induced by c-AMP) domain and C-terminus BID (*B**artonella* intracellular delivery) domain of BepC. The FIC domain played a key role in BepC-induced stress fiber formation and cell fragmentation because deletion of FIC signature motif or mutation of two conserved amino acid residues abolished BepC-induced cell fragmentation. Immunoprecipitation confirmed the interaction of BepC with GEF-H1 (a microtubule-associated RhoA guanosine exchange factor), and siRNA-mediated depletion of GEF-H1 prevented BepC-induced stress fiber formation. Interaction with BepC caused the dissociation of GEF-H1 from microtubules and activation of RhoA to induce formation of stress fibers. The ROCK (Rho-associated protein kinase) inhibitor Y27632 completely blocked BepC effects on stress fiber formation and cell contractility. Moreover, stress fiber formation by BepC increased the stability of focal adhesions, which consequently impeded rear-edge detachment. Overall, our study revealed that BepC-induced stress fiber formation was achieved through the GEF-H1/RhoA/ROCK pathway.

## Author summary

Intracellular pathogens modulate host cell actin cytoskeleton by secreting an array of effector molecules to ensure their cell invasion and intracellular survival. The zoonotic pathogen *Bartonella spp* trigger massive F-actin polymerization of host cells resulting the internalization of large bacterial aggregates (called “invasome” structure), which is dependent on a functional VirB/VirD4 type IV secretion system (T4SS) and its translocated Bep effector proteins. Here, we have used cell infection and ectopic expression assay to identify that *Bartonella* T4SS effector BepC induces stress fiber formation in infected host cells. However, BepC also disrupts the balance of stress fiber formation and focal adhesion maturation, and eventually causes cell fragmentation. Using immunoprecipitation and RNAi approaches, we identify GEF-H1 is the host factor targeted by BepC. Interaction with BepC induces the release of GEF-H1 from microtubules to plasma membrane and subsequently activates RhoA-ROCK to induce stress fiber formation. These findings shed light on our understanding of how *Bartonella* invade host cell and establish infection.

## Introduction

*Bartonella* species are facultative intracellular pathogens that are highly adapted to their specific mammalian hosts and vector reservoirs [1,2]. *Bartonella* colonizes alimentary tracts of lice or fleas, then forms a life-long commensal relationship [3]. Arthropods excrete *Bartonella* in their feces during feeding, and feces containing *Bartonella* bacteria are inoculated into skin lesions through scratching [4]. Subsequently, *Bartonella* penetrates the epithelial barrier via destruction of the tight junction of epithelial cells, and then hijacks host dendritic cells as a Trojan horse to disseminate *Bartonella* from the inoculation site [5,6]. Finally, *Bartonella* combats the phagocytic and pro-inflammatory effects of macrophages in draining lymph nodes and eventually invades the bloodstream through lymphatic circulation [7].

*Bartonella* harbors a VirB type IV secretion system (T4SS) that comprises 10 essential components (VirB2-VirB11) and a functionally associated coupling protein, VirD4 [8]. The VirB system translocates a cocktail of evolutionarily related *Bartonella* effector proteins (Beps) into host cells [9]. Beps are multi-domain proteins that mainly possess an N-terminal FIC (filamentation induced by c-AMP) domain that confers posttranslational modifications (PTMs) to substrates in host cells, and a C-terminal BID (*B**artonella* intracellular delivery) domain that acts as a signal for T4SS translocation. Some Beps lack a FIC domain but harbor tandem-repeated tyrosine motifs that is phosphorylated by host kinases, which subsequently interact with host SH2 domain proteins [10]. A recent study confirmed that tyrosine-phosphorylated BepD recruits STAT3 and c-Abl and induces STAT3 activation, which promotes the switch from pro- to anti-inflammatory response [11]. Besides, versatile functions of Beps had been identified, including inhibition of apoptosis, bacterial uptake via rearrangement of the F-actin cytoskeleton, activation of cell autophagy, and modulation of cell migration of infected host cells [12–15]. Among these, BepC was reported to involve with “invasome” formation of *Bartonella* through modulation of F-actin cytoskeleton. Moreover, BepE ensures the migration of dendritic cells to deliver *Bartonella* from derma to the bloodstream because BepE antagonizes BepC-induced host cell cytotoxicity [6]. This cytotoxic effect is characterized as disturbance of rear-edge detachment during the migration of infected migratory cells, and such cells become elongated and finally fragmented. However, the mechanism how BepC modulates F-actin cytoskeleton and subsequent cell fragmentation, remains elusive.

In this study, we identified that BepC exploited a guanine nucleotide exchange factor (GEF) of Rho GTPase, GEF-H1, to induce stress fiber formation and maturation of focal adhesion. The unbalance of contractile stress fiber formation and disassembly of focal adhesion upon translocation of BepC eventually caused cell fragmentation.

## Results

### BepC causes excessive stress fibers and a contractile cell morphology

**Fig 1:**
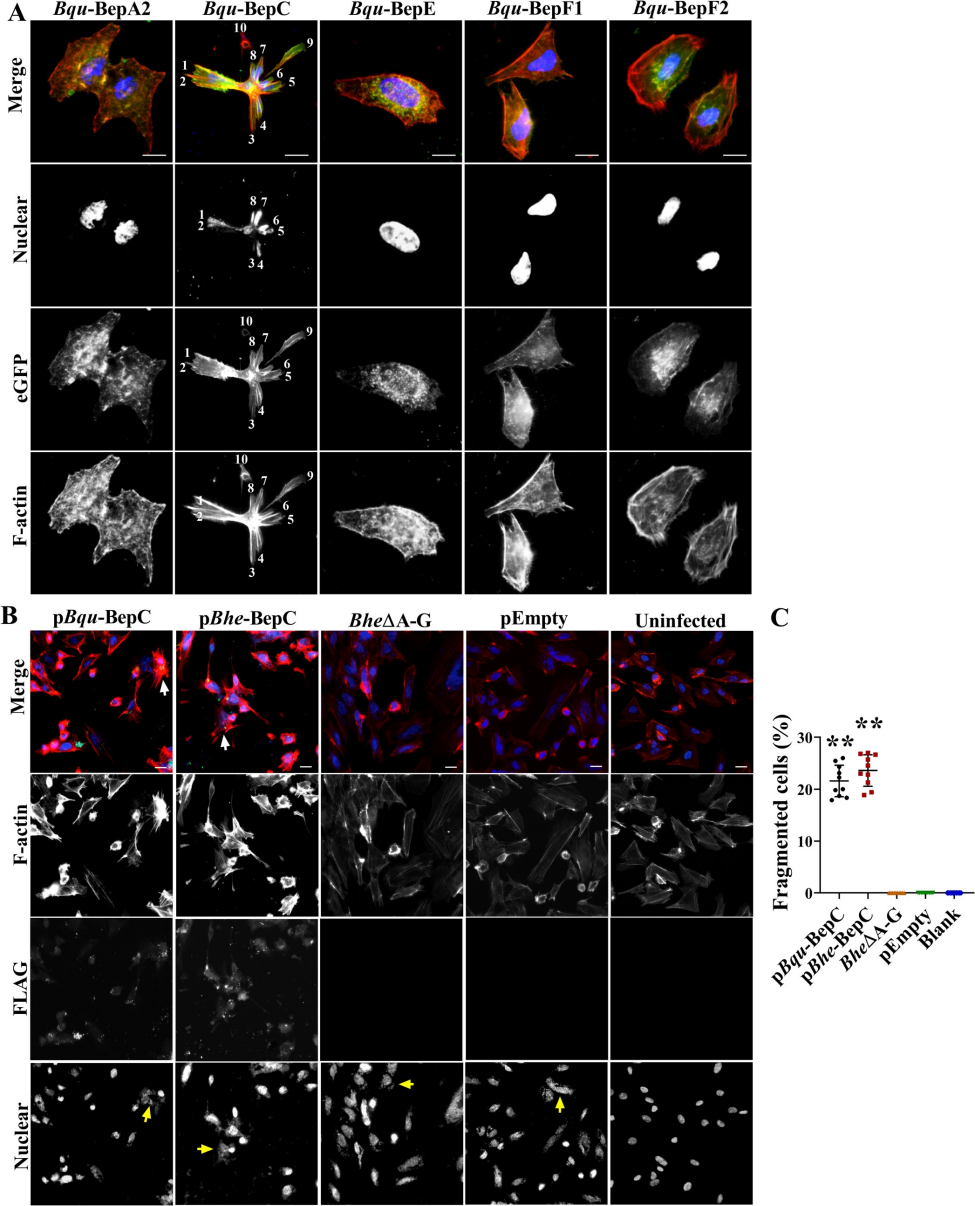
*Bqu*-BepC causes stress fiber formation and cell fragmentation. (A) Ectopic expression of all Bqu T4SS effectors tagged with eGFP, BepA2, BepC, BepE, BepF1 and BepF2, was investigated in transfected Hela cells. Stress fibers were stained with TRITC-phalloidin, and cell nuclei were stained with DAPI. Numbers represented connected pseudo-cells within a fragmented cell. (B) Morphological changes in HUVECs infected with Flag-BepC complemented strains with Bep locus deletion were identified. Stress fibers were stained with TRITC-phalloidin, FLAG-BepC was visualized by AlexaFluor 488 label secondary antibody and cell nuclei and bacteria were visualized by DAPI. The white arrow indicated the fragmented cells. The yellow arrow showed *Bartonella* bacilli. (C) Cells showing fragmentation were counted (infected cells in ten randomly selected visual fields were calculated). One-way ANOVA with multiple comparisons test was used. “**” p < 0.001. All experiments were performed more than three times independently, and representative data are shown. Values shown are means ± SD. Bar = 10 μm.

Firstly, we were interested to investigate if cell fragmentation induced by *Bartonella quintana* (*Bqu*) is induced by single effector. Five individual effectors from *Bqu*, BepA, BepC, BepE, BepF1 and BepF2, were ectopically expressed in HeLa cells. Among these, only the expression of BepC caused significant changes in cell morphology, which were characterized by formation of excessive stress fibers leading to a contractile morphology towards the center of cell bodies (Fig 1A). Some daughter cell bodies (white arrows) were even separated from their parent cells (S1 Movie). These features resembled the observation in a previous study that small portions were left behind from a given cell body during cell migration after infection with a *BepE* deleted *Bartonella henselae* strain [6]. In addition, *Bqu*-BepC induced significant segmentation of cell nuclei, with each segmented nucleus located in a fragmenting cell body forming connected pseudo-cells (Fig 1A). Although fragmented cells were not undergoing necrotic cell death or apoptosis, this process led to cytotoxicity and decreased cell number (S1 Fig). Furthermore, such fragmentation was not cell-type specific since the transfection of *Bqu*-BepC in HUVECs, HEK293T and even mouse MEFs (mouse embryonic fibroblast) all caused cell fragmentation (S2 Fig). Previous studies noted the functional conservation of certain effector orthologues from different *Bartonella* species. Here, we also ectopically expressed the BepC from *Bartonella henselae* (*Bhe*) and *Bartonella tribocorrum* (*Btr*) in HeLa cells. As expected, all BepC induced stress fiber formation, while *Btr*-BepC was distinguished from those two orthologues showing less ability to induce cell fragmentation (S3 Fig). Subsequently, we used a *Bep* locus deletion strain of *Bhe* (Δ*BepA-G*) to infect host cells [14], along with complemented strains expressing *Bqu*-BepC, *Bhe*-BepC and empty vector control. As expected, only cells infected by the two BepC complementation strains exhibited significant formation of stress fibers and cell fragmentation. However, cell nucleus segmentation caused by T4SS-delivered *Bqu*-BepC was not frequently observed as occurred in ectopic expression (Fig 1B and 1C).

### Disturbance of focal adhesion disassembly by BepC results in cell fragmentation

**Fig 2:**
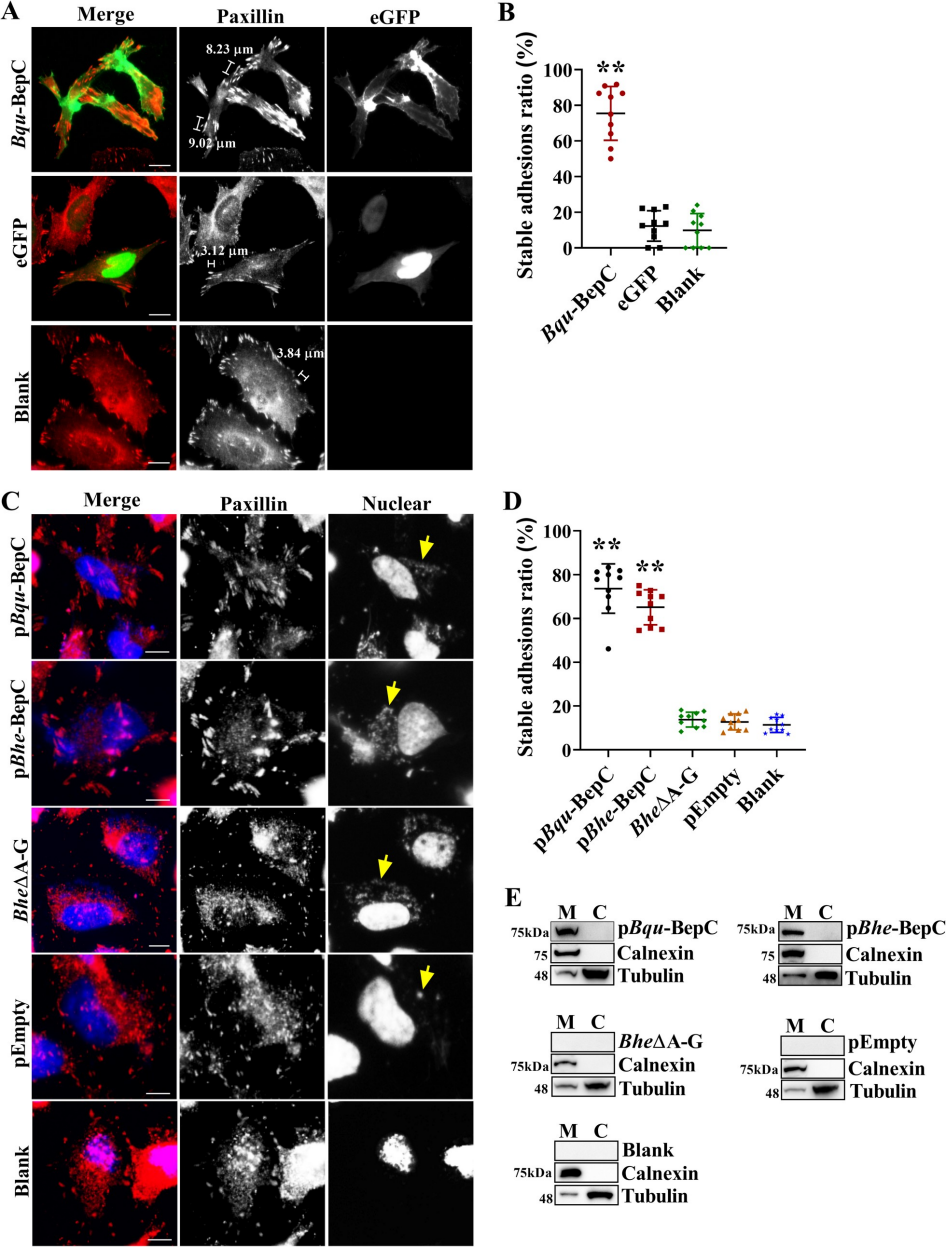
*Bqu*-BepC induces maturation of focal adhesion. (A) After expression of eGFP-BepC and control eGFP empty vector in Hela cells by transfection, focal adhesion was visualized by Paxillin staining. Stable focal adhesion was defined as a length of over 5 μm (scale bar indicated). Focal adhesions were visualized in red, green channel indicated the expressing of BepC protein. (B) The percentage of stable adhesions in total focal adhesions was calculated in transfected cells (transfected cells in ten randomly selected fields were calculated). One-way ANOVA with multiple comparisons test was used. “**” p < 0.001. (C) Maturation of focal adhesion in cells infected with *Bep* locus deletion and FLAG-BepC complemented *Bhe* strains was investigated by Paxillin immunostaining. Focal adhesions were shown in red and blue showed cell nucleus and bacteria. Yellow arrows indicated *Bartonella* bacilli. (D) The ratio of stable adhesions in total adhesions was calculated (infected cells in ten randomly selected view fields were calculated). One-way ANOVA with multiple comparisons test was used. “**” p < 0.001. (E) Fractions of cytosol and membrane of cells were prepared 36 h post infection. Immunoblot was performed to investigate the T4SS delivery of FLAG-tagged BepC. Calnexin and α-tubulin were used to specify cell membrane and cytoplasmic fraction. “M” indicated membrane and “C” indicated cytosol. Representative data of 3 independent experiments are shown. Values shown are means ± SD. Bar = 10 μm.

A live cell image assay showed that polarized migration of BepC expressing cells resulted in dragging of trailing tails and elongation (S1 Movie). Based on this, it was proposed that BepC impeded detachment of the rear edge and disrupted its coordination with cell contractility formation. It is known that the contractile actin stress fiber assembly is important for the maturation of focal contacts into larger focal adhesions (FAs) at the leading edge, but promotes disassembly of FAs at the rear edge [16]. Therefore, we investigated the effects of BepC on focal adhesion maturation. Here, mature focal adhesion was defined as a length of over 5 μm as reported in previous studies [17,18]. Our results confirmed that ectopic expression of BepC significantly increased the number of mature focal adhesions in comparison with eGFP-expressed controls. Cell contractility towards the cell center after BepC expression pulled the focal adhesions (Fig 2A and 2B). Infection with *BepC* complemented Δ*BepA-G* mutant strains also confirmed the maturation of focal adhesions (Fig 2C and 2D). T4SS translocation of BepC was further investigated by immunoblots after 36 h infection. Results confirmed BepC was successfully translocated into host cells by two complementation strains. Interestingly, BepC was found to localize within fraction of plasma membrane (Fig 2E). Taken together, these data indicated that BepC broke the fine balance of cell adhesion and cell contractility, leading to cell fragmentation.

### BepC-induced cell fragmentation requires both the N-terminal FIC and C-terminal BID domains

**Fig 3:**
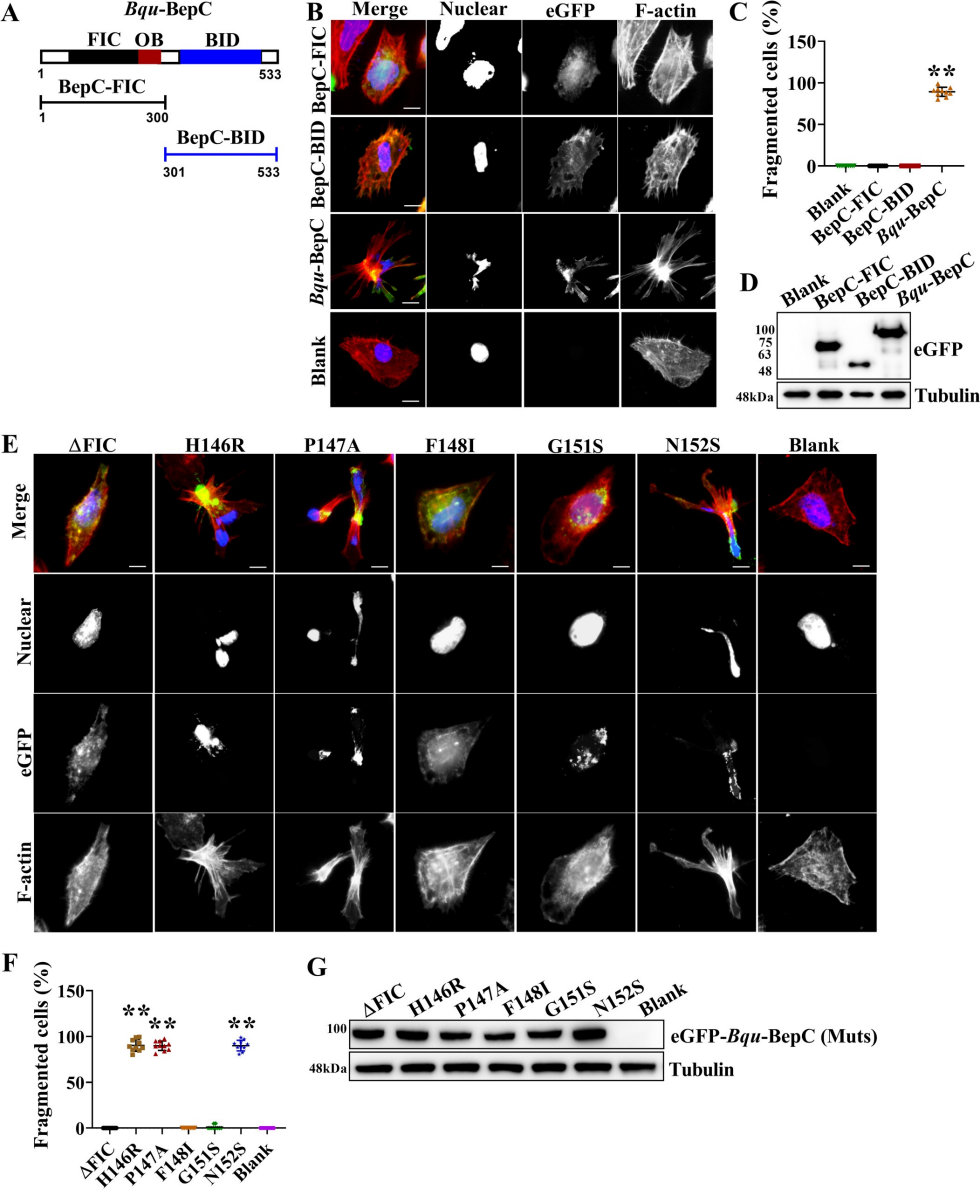
Both the FIC (filamentation induced by c-AMP) and BID (*Bartonella* intracellular delivery) domain are required for BepC-induced stress fiber formation. (A) Graphical depiction of the main domains of *Bqu*-BepC. (B) FIC and BID. Truncations, and full-length BepC tagged with eGFP were expressed in Hela cells by transfection. Stress fibers and cell nucleus were visualized by TRITC-phalloidin and DAPI. (C) Cells showing fragmentation after expression of full-length BepC, BID and FIC truncations were calculated (transfected cells in ten randomly selected fields were calculated). One-way ANOVA with multiple comparisons test was used. “**” p < 0.001. (D) Expression of eGFP-tagged protein was further detected by immunoblot. (E) FIC motif mutants, including signature motif deletion and site mutations, were transfected in Hela cells. ΔFIC represented the mutant with the deletion of signature motif (HPFxxGNG). Stress fibers were visualized by TRITC-phalloidin, the eGFP channel showed the expression of BepC and the corresponding mutants, and the cell nucleus were shown in blue. (F) Cell fragmentation caused by BepC mutants was calculated (transfected cells in ten randomly selected fields were calculated). One-way ANOVA with multiple comparisons test was used. “**” p < 0.001. (G) Expression of BepC and corresponding mutants was further confirmed by immunoblots. All assays were performed more than three times independently, and representative data are shown. Values shown are means ± SD. Bar = 10 μm.

BepC, like most other *Bartonella* VirB/VirD4 effectors, maintains FIC-BID architecture. Additionally, BepC contains an oligosaccharide binding (OB) fold between FIC and BID domains. The C-terminal BID domain together with a positively charged tail sequence constitutes the secretion signal for T4SS translocation, while the function of the N terminus FIC is currently unknown [19]. Here, two truncated mutants of *Bqu*-BepC were constructed, corresponding to either N-terminal FIC domain containing an OB fold or C-terminal BID domain along with a positively charged tail (Fig 3A). FIC proteins primarily catalyze the AMPylation of protein substrates (although other modifications such as UMPylation, GMPylation, phosphorylation and phosphocholination have been reported as well), leading to conformational changes that perturb the structure of the target protein and thus its function [20]. Here, neither individual FIC-OB nor BID truncation caused stress fiber formation and cell fragmentation (Fig 3B–3D). Next, we investigated the effect of point mutations of the FIC signature motif (HPF××GNG) on BepC-induced cell fragmentation. BepC function was not affected by mutation of the catalytic histidine (H146R), which is critical for all known FIC-mediated modifications [20]. Mutation of asparagine (N152S), known to hold the catalytic loop in its peculiar structure, and a less conserved amino acid proline (P147A) were also ineffective on BepC function [21]. Nevertheless, F148I mutation, which is known to anchor the catalytic loop to the hydrophobic core of the enzyme [21], and mutation of G151S, the first amino acid of the submotif, forms an ‘anion hole’ that favors interaction with the oligophosphate moiety of nucleotides [22], showed a determinant effect to stress fiber formation and cell fragmentation. Moreover, deletion of the FIC motif completely abolished BepC-induced cell fragmentation (Fig 3E–3G). Although enzymatic function of BepC was not evidenced in the present study, obtained data here indicated a potential contribution of FIC activity to BepC-induced cell fragmentation, possibly, though in a manner distinguishable from all known FIC functions.

### Interaction with GEF-H1 contributes to BepC-induced cell fragmentation

**Fig 4:**
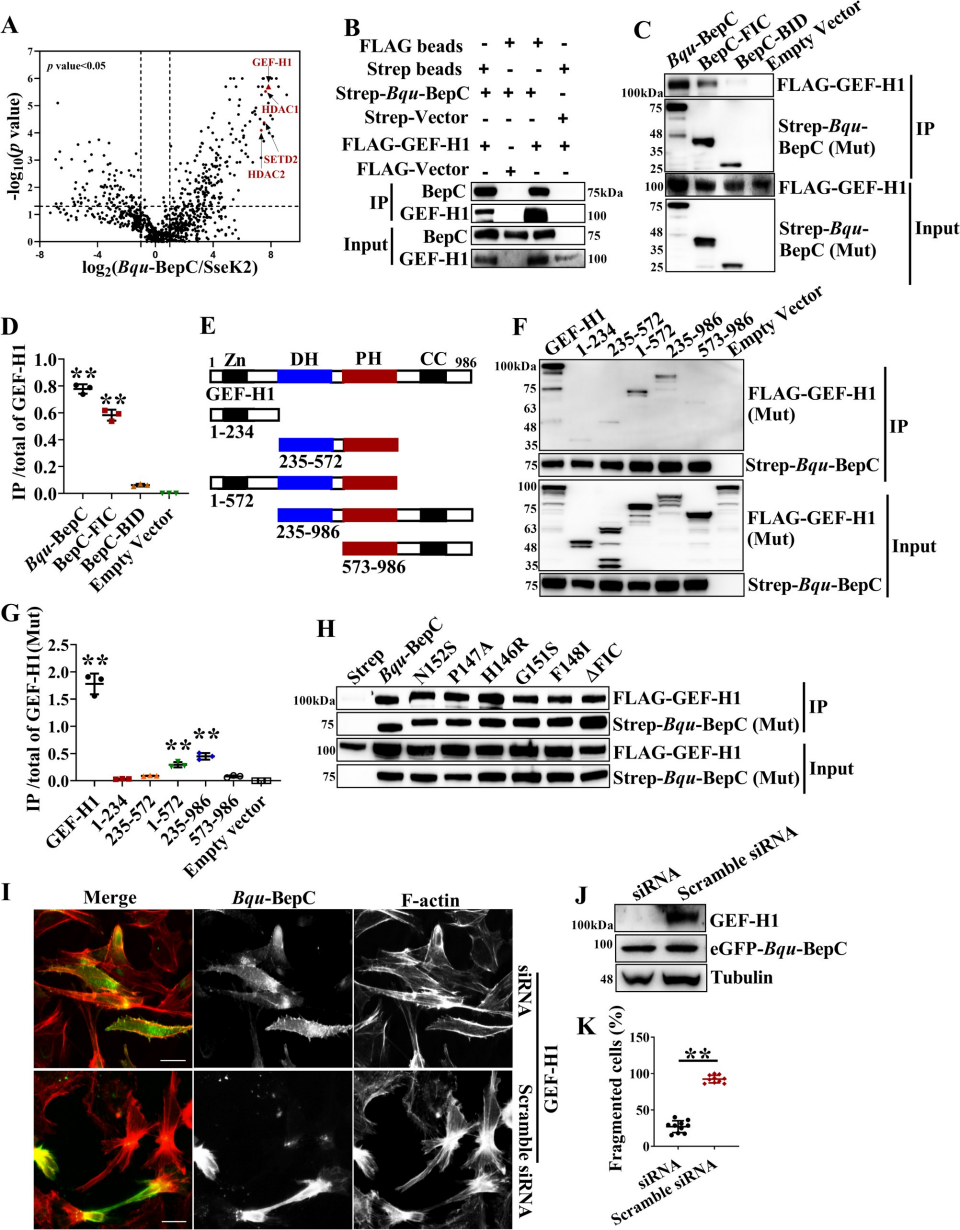
*Bqu*-BepC targets GEF-H1 to induce stress fiber formation. (A) Strep tagged BepC was purified by streptactin beads from transfected HEK293T cells, and then potential binding candidates for BepC were identified by LC-MS/MS. The highly specific candidates of BepC in the host cell (*Salmonella* effector SseK2 served as control) are illustrated in Volcano plots. (B) Strep-BepC and FLAG-GEF-H1 were co-transfected in HEK293T cells, and then co-immunoprecipitated by indicated affinity beads (Strep beads indicated the immunoprecipitation of Strep tagged proteins, Flag beads indicated the immunoprecipitation of flag tagged proteins). After that, immunoprecipitated samples were detected by immunoblot. (C) Co-transfection of Strep tagged FIC, BID truncations and full-length BepC with FLAG tagged GEF-H1 was performed in HEK293T cells, co-immunoprecipitation was performed by using streptactin beads. After that, immunoprecipitated samples were detected by immunoblotting. The empty vector served as a negative control. (D) Binding of full-length BepC, BID and FIC with GEF-H1 was analyzed by calculation of band intensity to indicate the proportion of GEF-H1 within co-immunoprecipitated BepC and its truncated versions in comparison to empty vector. One-way ANOVA with multiple comparisons test was used. “**” p < 0.001. (E) Graphic demonstration of GEF-H1 protein structure. (F) Domain truncations of GEF-H1 fused with FLAG tag and Strep-BepC were co-transfected in HEK293T and were subjected to co-immunoprecipitation using streptactin beads, and their interaction was also explored by immunoblots. (G) The binding efficiency of full-length GEF-H1 and its domain truncations was analyzed by calculation of band intensity to indicate the proportion of GEF-H1 and its truncations within co-immunoprecipitated BepC in comparison with empty vector. One-way ANOVA with multiple comparisons test was used. “**” p < 0.001. (H) Binding of FIC mutants of BepC with co-transfected GEF-H1 was also investigated by Co-IP with streptactin beads as mentioned above. (I) Endogenous GEF-H1 was depleted by siRNA in stable expression cells. Subsequently, expression of eGFP-BepC in treated cells were induced by doxycycline. Stress fiber was visualized by TRITC-phalloidin. (J) Depletion of endogenous GEF-H1 by siRNA was confirmed by immunoblot. (K) The percentage of fragmented cells (eGFP-BepC positive cells in 10 randomly selected visual fields were calculated) was analyzed in the GEF-H1 depletion and control group. Student’s t test was used. “**” p < 0.001. All assays were performed more than three times independently and representative data are shown. Values shown are means ± SD. Bar = 10 μm.

Stress fiber formation and focal adhesion assembly are precisely regulated by number of small GTPases and their regulators. We reasoned that BepC might hijack one or some of these key factors to promote the formation of stress fiber. To identify the binding substrates of BepC in host cells that contributed to stress fiber formation and cell fragmentation, immunoprecipitate of BepC was collected and subjected to LC-MS/MS analysis. Interestingly, GEF-H1, a Dbl family guanine nucleotide exchange factor of Rho and Rac GTPase [23], was detected to be specifically associated with BepC (Fig 4A and S1 Table). Forward and reverse immunoprecipitation from co-transfected cells confirmed the interaction of BepC with GEF-H1 (Fig 4B). Furthermore, co-immunoprecipitaion *in vitro* by using purified BepC from transfected 293T (because prokaryotic expression of BepC is not soluble) and GST tag fused GEF-H1 from *E*. *coli* verified the interaction of BepC with GEF-H1 (S4 Fig). Although above-mentioned data showed that only full-length BepC caused stress fiber formation and cell fragmentation (Fig 3B), we found that truncated FIC domain containing a OB fold interacted with GEF-H1 in host cells, while BID domain showed no binding activity with GEF-H1 (Fig 4C and 4D). GEF-H1 is composed of an N-terminal zinc finger domain, a Dbl-homologous (DH) domain followed by a Pleckstrin homology (PH) domain, and a C-terminal domain [24] (Fig 4E). The zinc-finger domain and C-terminal domain are involved in the association of GEF-H1 with microtubules and maintain GEF-H1 in an inactive state [25]. Here, we confirmed that the C-terminus containing the DH-PH domain and N-terminus containing the DH-PH domain were co-immunoprecipitated with BepC, while individual C-terminus, N-terminus and DH-PH domains showed weak binding to BepC (Fig 4F and 4G). We reasoned that the DH-PH domain served as the binding site for BepC only if it associated with microtubule when combined with the C-terminus or N-terminus. Although F148I, G151S and FIC motif deletion mutants failed to induce stress fiber formation, binding with GEF-H1 was still identified in co-transfected cells (Fig 4H). Subsequently, siRNA-mediated knock-down of GEF-H1 expression was established in HeLa cells. BepC-induced stress fiber formation was significantly inhibited in GEF-H1 siRNA treated cells (Fig 4I and 4J), and the number of cells showing contractility or fragmentation decreased substantially in comparison to control siRNA treated cells (Fig 4K).Taken together, the observations demonstrated that the interaction of GEF-H1 and BepC contributed to BepC-induced cell fragmentation.

### BepC causes the dissociation of GEF-H1 from microtubules

**Fig 5:**
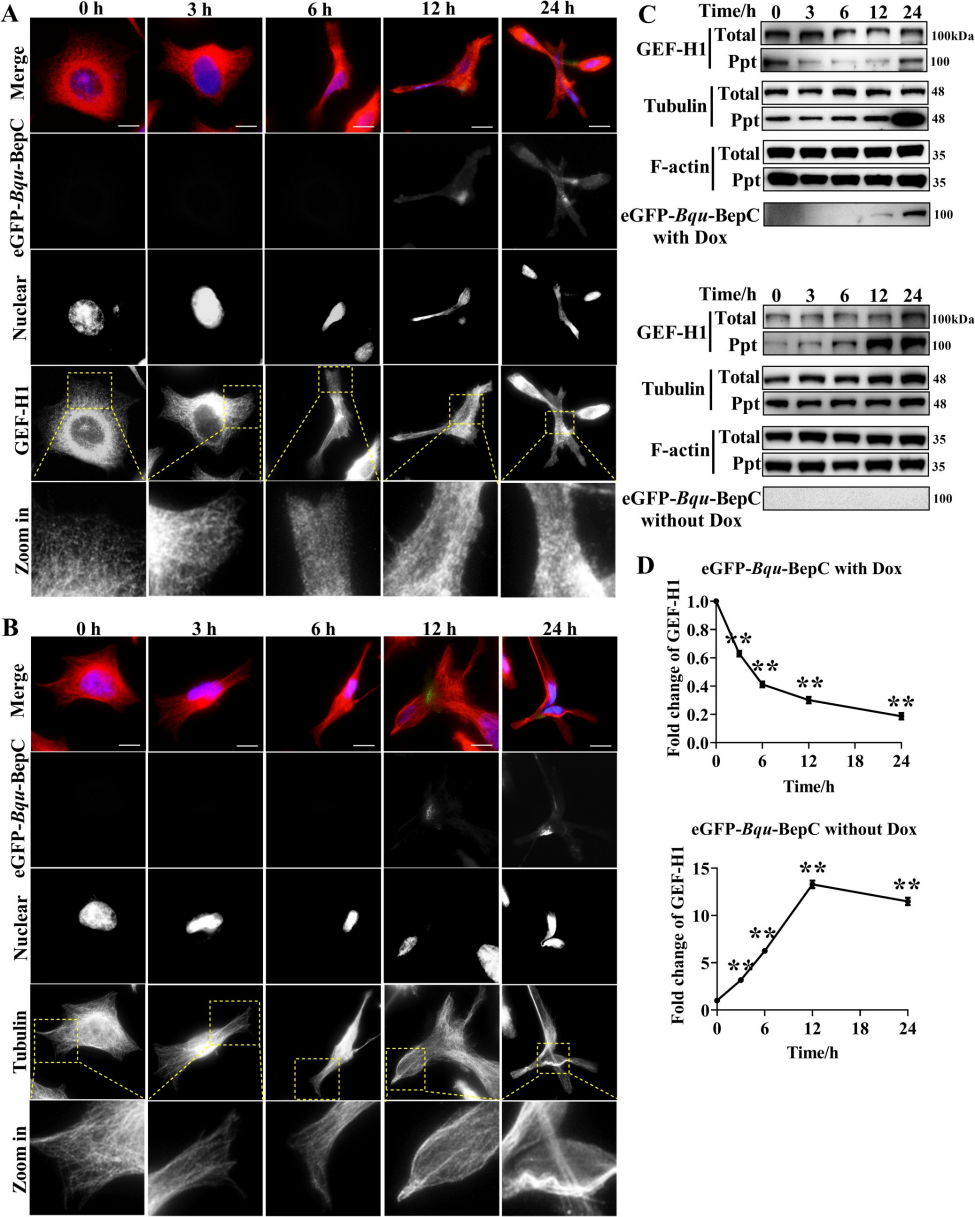
*Bqu*-BepC induces the dissociation of GEF-H1 from microtubules. (A) Expression of BepC with eGFP tag was induced by adding doxycycline in stable cell line. At indicated time points after doxycycline treatment, endogenous GEF-H1 was probed with GEF-H1 antibody (in red). eGFP-BepC was shown in green channel and cell nucleus were visualized by DAPI staining. (B) Accordingly, microtubules were visualized by anti-tubulin antibody at the corresponding time points. Yellow boxes denoted the area used for the insert. (C) At corresponding time point after doxycycline induction, cytoskeleton-enriched fraction was collected. Localization of GEF-H1 in cytoskeleton fraction was investigated by immunoblots. Cells without doxycycline induction were used for negative control. Ppt indicated cellular precipitation fraction. (D) Protein intensity of GEF-H1 in precipitation fraction (normalized with α-tubulin) at different time points was quantified as the fold change of initial GEF-H1 level in cytoskeleton fraction. One-way ANOVA with multiple comparisons test was used. “**” p < 0.001. All assays were performed more than three times independently, and representative data are shown. Values shown are means ± SD. Bar = 10 μm.

**Fig 6:**
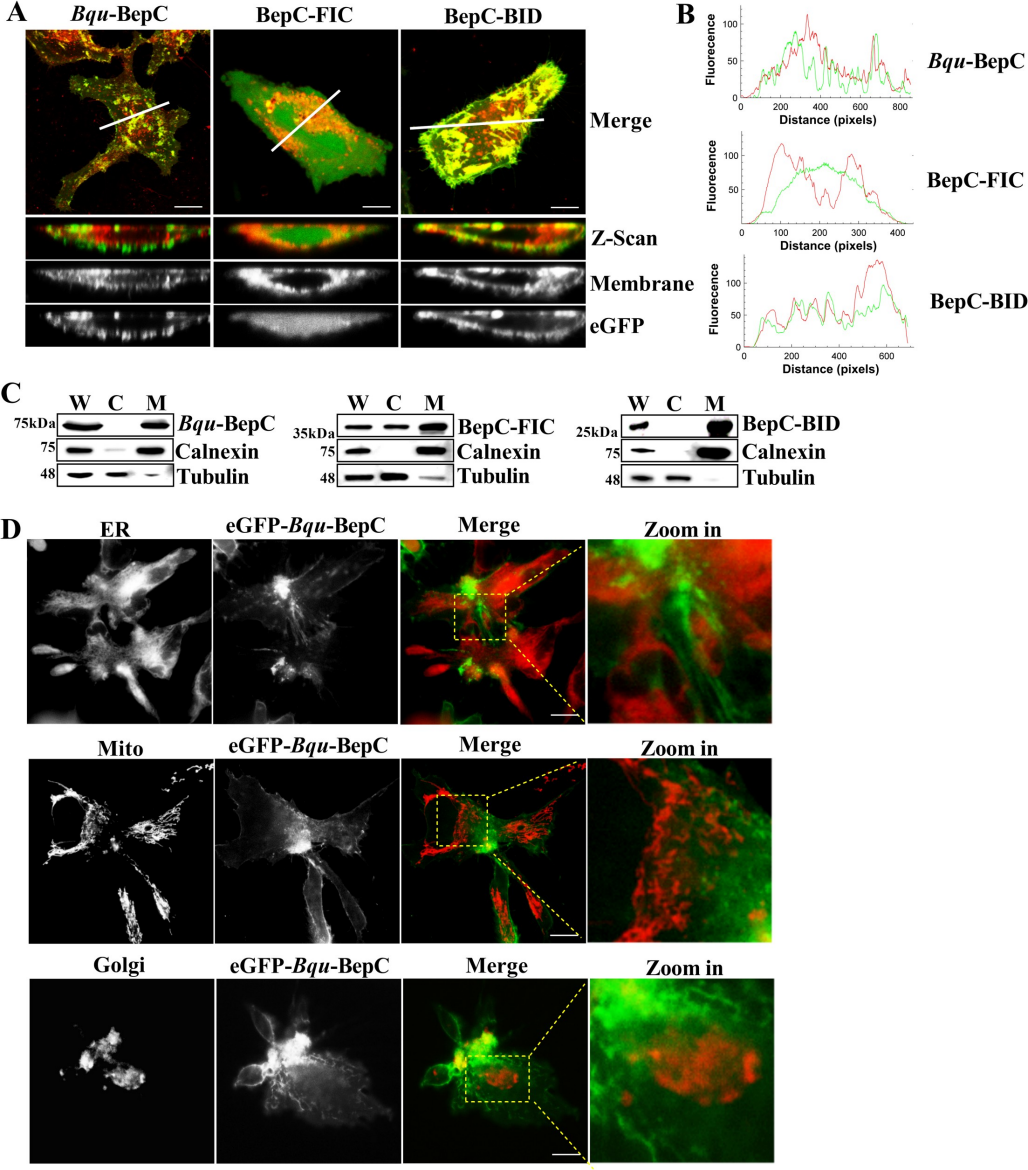
*Bqu*-BepC localized within plasma membrane. (A) Full-length BepC, truncated version of FIC and BID with eGFP tag were ectopically expressed in Hela cells by transfection, and the localization was investigated by immunofluorescence using a Z-scan. Plasma membrane was stained with a membrane probe (DiIC18(3), red). (B) Plot profile of Z-scan images generated by ImageJ was used to determine the co-localization of BepC and plasma membrane. (C) HEK293T cells were transfected with Strep tagged full-length BepC, and two truncations of FIC and BID domain. Cellular fractionation was prepared. Cellular localization of Strep tagged BepC and its truncated versions was further investigated by immunoblots. Tubulin and calnexin were used to specify cytosol and cell membrane localization, respectively. W: whole cell lysate, C: cytosol, M: plasma membrane. (D) Localization of BepC with specific organelle, including endoplasmic reticulum, golgi apparatus and mitochondria, was further investigated by immunofluorescence assay. Red colour indicated organelle, and green indicated transfected BepC. All assays were performed more than three times independently, and representative data are shown. Bar = 10 μm.

GEF-H1 plays important roles in regulating the coordination between microtubules (MTs) and the actin cytoskeleton. GEF-H1 is sequestered in an inhibitory state on the microtubule, which in turn benefits the stability of the microtubule meshwork. Alternatively, GEF-H1 can induce stress fiber formation upon its dissociation from the microtubule [25]. Therefore, we explored the effects of interaction with BepC on GEF-H1 localization. Here, we confirmed expression of BepC induced transformation of the meshwork of GEF-H1 to a diffused pattern, even at very early stages when BepC was not detectable by immunoblots (Fig 5A). It is known that dissociation of GEF-H1 impedes the stability of microtubules [26]. Therefore, it was instructive for us to further investigate the microtubule network after GEF-H1 dissociation. However, immunostaining of endogenous GEF-H1 by the antibody we used required cold methanol fixation, which is not well compatible with tubulin staining. Accordingly, these two proteins were immunostained separately. Interestingly, thick tubulin bundles were frequently observed in BepC expressed cells after 24 h induction, which made MTs much brighter than that in previous time points. However, systematical MTs enclosure of the cell nucleus was impaired, which was in accordance with our observation that BepC expression decreased host cell rigidity and caused cell nucleus bulging (Fig 5B and S1 Movie). To further demonstrate the release of GEF-H1 from the tubulin cytoskeleton, the cytoskeleton-enriched protein fraction was isolated and investigated by using immunoblots. In control cells (without doxycycline induction), increasing localization of GEF-H1 to microtubule was identified during cell proliferation. However, the expression of BepC decreased the level of GEF-H1 in cytoskeleton fraction (Fig 5C and 5D), while increase of GEF-H1 in MTs was confirmed (it might be explained that increase number of cells in interphase during cell proliferation will increase the localization of GEF-H1 in microtubule). Notably, significant enrichment of tubulin into cytoskeleton fraction was identified after 24 h expression of BepC. Together with the finding from immunofluorescent experiments, it indicated BepC can induce the repolymerization of microtubule. Nevertheless, F-actin level in cytoskeleton fraction was unchangeable during BepC expression although significant stress fiber formation was identified by immunofluorescent assay. This finding indicated that BepC mainly induced the formation of ventral stress fiber that were formed from the pre-existing dorsal stress fibers (instead of a denovo polymerization of actin filaments). Using xzy-plane scanning immunofluorescence assay, we identified a peripheral localization of the full-length BepC and C-terminus BID domain in the host cell, while the FIC domain showed a dispersed localization in the cytosol (Fig 6A). Plot profile analysis indicated a co-localization of BepC and BID with plasma membrane (Fig 6B). We further investigated the cellular localization of ectopically expressed BepC and its truncated versions in cells by immunoblotting of cellular fraction. As expected, the results showed that full-length BepC and BID truncation localized within the membrane fraction, and localization of FIC truncation shifted from membrane fraction to cytosol (Fig 6C). In viewing that organelle membrane was also collected with plasma membrane by the experiment settings used here, localization of BepC with specific organelle was further analyzed by using immunofluorescence assay. Results showed no co-localization of BepC with endoplasmic reticulum, Golgi apparatus and mitochondria (Fig 6D). Thus, the co-localization of BepC with plasma membrane was demonstrated. Altogether, our data indicated both FIC-OB-mediated GEF-H1 binding and BID-mediated plasma membrane localization are essential for BepC-induced stress fiber formation and cell fragmentation.

### BepC promotes stress fiber formation via the GEF-H1/RhoA/ROCK pathway

**Fig 7:**
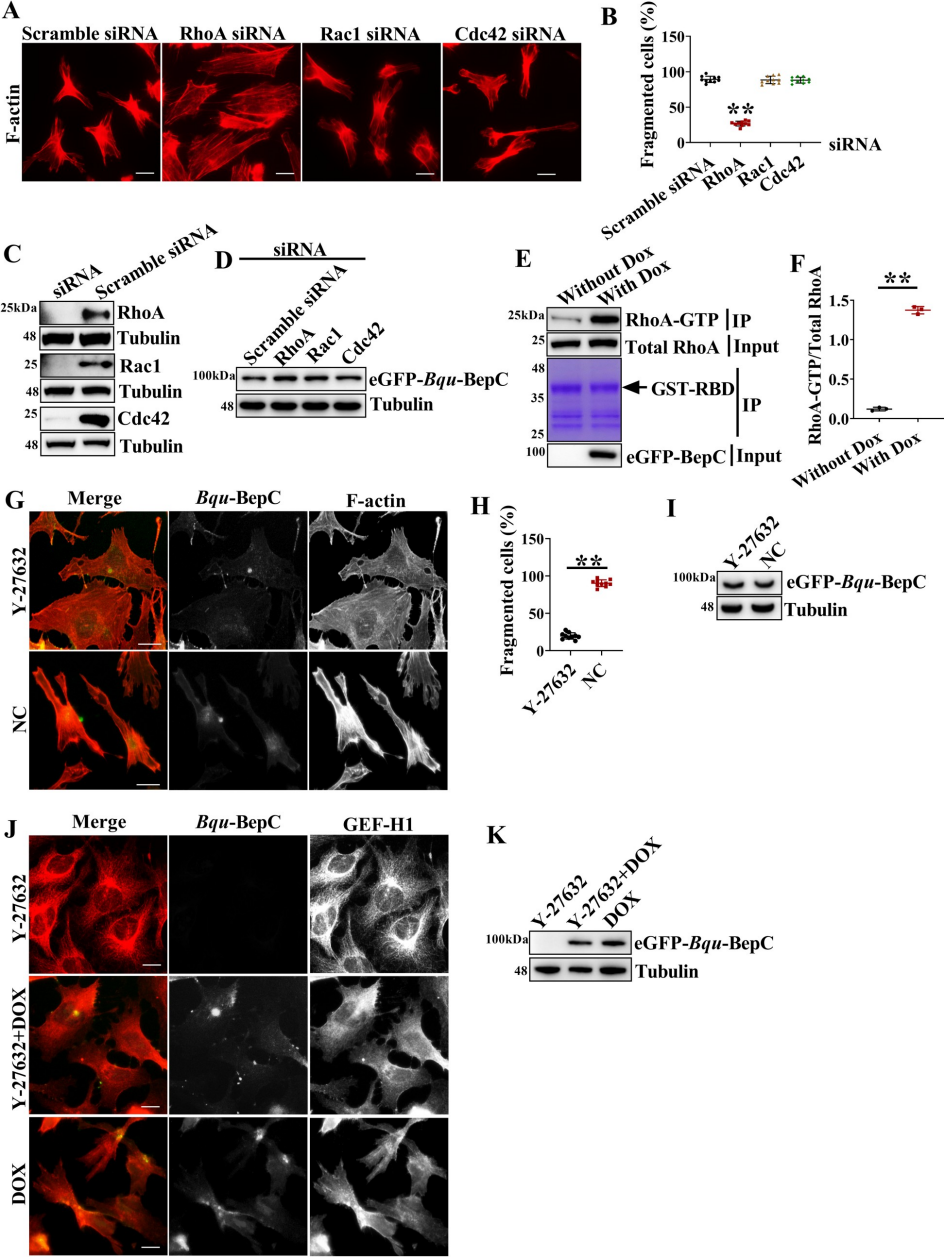
*Bqu*-BepC hijacks the GEF-H1/RhoA/ROCK pathway to induce stress fiber formation. (A) After depletion of RhoA, Rac1 and Cdc42, BepC expression was induced by adding doxycycline, and then, stress fibers were probed with phalloidin. Scramble siRNA was used as negative control. (B) Percentage of cells showing fragmentation in siRNA treated cells was calculated (cells in ten randomly selected visual fields were calculated). One-way ANOVA with multiple comparisons test was used. “**” p < 0.001. (C) Endogenous RhoA, Rac1 and Cdc42 were depleted individually in stable expression cells by siRNA, and expression of these proteins was detected by immunoblots. (D) Expression of eGFP-tagged BepC induced by doxycycline in the corresponding cells (siRNA treatment as above mentioned) was further investigated by immunoblot. (E) GTPase activity of RhoA was investigated by GST-RBD binding assay in cells with or without doxycycline induction. BepC expression and total RhoA was detected in whole cell lysate (input), equal loading of GST-RBD was confirmed by Coomassie blue staining of GST immunoprecipitation samples. GTP-bound RhoA was detected in Co-IP sample. (F) Protein intensity was calculated to indicate the relative proportion of GTP bound RhoA with total RhoA in cells with or without doxycycline induction. Student t test was used. “**” p < 0.001. (G) ROCK inhibitor Y-27632 was added before BepC induced, and then, stress fibers were visualized with phalloidin staining (red) after BepC (green) was induced after 24 h. NC indicated no drug treatment. (H) Percentage of cells showing fragmentation in ROCK inhibitor Y-27632 treated cells was calculated (cells in ten randomly selected visual fields were calculated). Student t test was used. “**” p < 0.001. (I) Expression of eGFP-tagged BepC induced by doxycycline in stable cells was further investigated by immunoblot. (J) ROCK inhibitor Y-27632 treated cells were probed with GEF-H1 antibody (red), BepC in green. (K) Expression of BepC was detected by immunoblot. Data from one representative experiment data (n = 3) were shown. Values shown are means ± SD. Bar = 10 μm.

GEF-H1 is a guanine nucleotide exchange factor that can activate RhoA and Rac1 [23]. PAK4-dependent phosphorylation on GEF-H1 acts as a switch that blocks GEF-H1-dependent stress fiber formation and promotes Rac1-dependent lamellipodia generation in fibroblasts [27]. Based on the phenotype that BepC induced excessive stress fiber formation, it was highly possible that modulation of the actin cytoskeleton by BepC was also RhoA dependent. In the present study, three Rho GTPases, RhoA, Rac1 and Cdc42, were down regulated by siRNA. As expected, siRNA-mediated depletion of RhoA significantly blocked the formation of stress fiber in BepC inducible expression cells, while down regulation of Rac1 and Cdc42 did not impact BepC function (Fig 7A–7D). To validate the relevance of BepC function with RhoA, the Rho binding domain (RBD) of the Rhotekin protein was expressed as a GST-fusion protein in *E*. *coli*. RBD binding assay also confirmed that RhoA was highly activated after BepC expression (Fig 7E and 7F). RhoA acts through the downstream effector Rho-associated kinase (ROCK) to induce the assembly of stress fiber and cell contractility [28,29]. Here, ROCK inhibitor Y27632 effectively decreased the BepC-induced stress fiber and cell fragmentation (Fig 7G–7I), although GEF-H1 was dissociated from the microtubules (Fig 7J and 7K). Taken together, these data suggest that BepC-induced cell fragmentation was achieved through hijacking the GEF-H1/RhoA/ROCK pathway.

## Discussion

The actin cytoskeleton is an essential component of all eukaryotic cells, which controls or mediates cell migration, cell shape, cell division and intracellular membrane trafficking through actin polymerization and depolymerization [30–33]. Therefore, the actin cytoskeleton and its regulators are major targets of bacterial pathogens for invasion, inhibition of phagocytosis, intracellular motility and cell-to-cell spread [34–36]. Bacteria have evolved a large arsenal of toxins and effectors, which are mainly injected into host cells through secretion systems (T3SS, T4SS, T5SS) [37,38]. Bacterial toxins or effectors affect the actin cytoskeleton by modifying actin directly or mimic and functionally override some regulator functions [34,35,39]. Among these, Rho GTPases are most commonly found to be modulated by bacterial pathogens, leading to the activation or inhibition of GTPase function corresponding to actin cytoskeleton rearrangements, either through enzymatic post-translation modifications (ADP-ribosylation, AMPylation, N^ɛ^-Fatty acylation) [40–42] or by mimicking host regulators of Rho GTPase activity (GEF or GAP function) [43–46].

A previous study reported that the *Bartonella* T4SS effector BepC, together with BepF, facilitated the internalization of a large bacterial aggregate (invasome) in host cells by modulating actin cytoskeleton polymerization, after that, BepC triggers host cell fragmentation in absence of BepE [6,14]. In this study, we found that BepC activated GEF-H1 by releasing it from MTs, which in turns to activate RhoA/ROCK pathway to induce the formation of stress fibers in infected host cells (see graphical abstract). GEF-H1 is a microtubule-associated guanine nucleotide exchange factor that activates RhoA or Rac1 upon release from microtubules. GEF-H1 was reported to undergo complicated regulation by phosphorylation at 36 different residues. For instance, phosphorylation of GEF-H1 on site Ser^885^ by PAK1 and PKA inactivated GEF-H1 by associating it with polymerized microtubules when 14-3-3 was bound to phosphoSer^885^ in the C-terminus [47,48], while phosphorylation on Ser^151^ by MARK3 triggered the release of GEF-H1 from the microtubule and stabilized it in an active state when 14-3-3 was bound to phosphoSer^151^ in the N-terminus [49]. In this study, FIC domain was shown to be essential for BepC-induced stress fiber formation. However, histidine mutation (the critical residue for catalysis of AMPylation) did not interfere with the phenotype triggered by BepC, which indicated BepC does not act as a canonical AMPylator. Although mutation of two sites in signature motif known to anchor the catalytic loop and oligophosphate moiety of nucleotides abolished BepC-mediated cell fragmentation, our data evidenced that these mutants still maintained the ability to bind with GEF-H1. Therefore, whether BepC harbors a novel FIC function by using substrate other than ATP to modulate GEF-H1 and results its dissociation from microtubule, is worthy to be investigated in future.

Cell migration is spatiotemporally regulated under the fine balance of stress fiber formation and focal adhesion dynamics [50–52]. RhoA-mediated stress fibers are known to drive focal adhesion assembly at the leading edge, but promote adhesion disassembly at the trailing edge, causing cell rear detachment [28,52,53]. BepC induces contractile stress fiber formation, but stabilizes focal adhesions, causing the unsuccessful detachment of the rear edge in a synchronized fashion. Disruption of the fine balance of actin-based contractility and FA disassembly explains how BepC induces cell fragmentation. Other effectors, namely, EspG and EspG2 from Enteropathogenic *E*. *coli* and VopO from *Vibrio parahaemolyticus*, were also reported to induce stress fiber formation by depolymerizing microtubule in consequence to activate GEF-H1 or directly activating GEF-H1, respectively [29,54]. However, no stabilization of focal adhesion and subsequent cell fragmentation were recorded.

**Fig 8:**
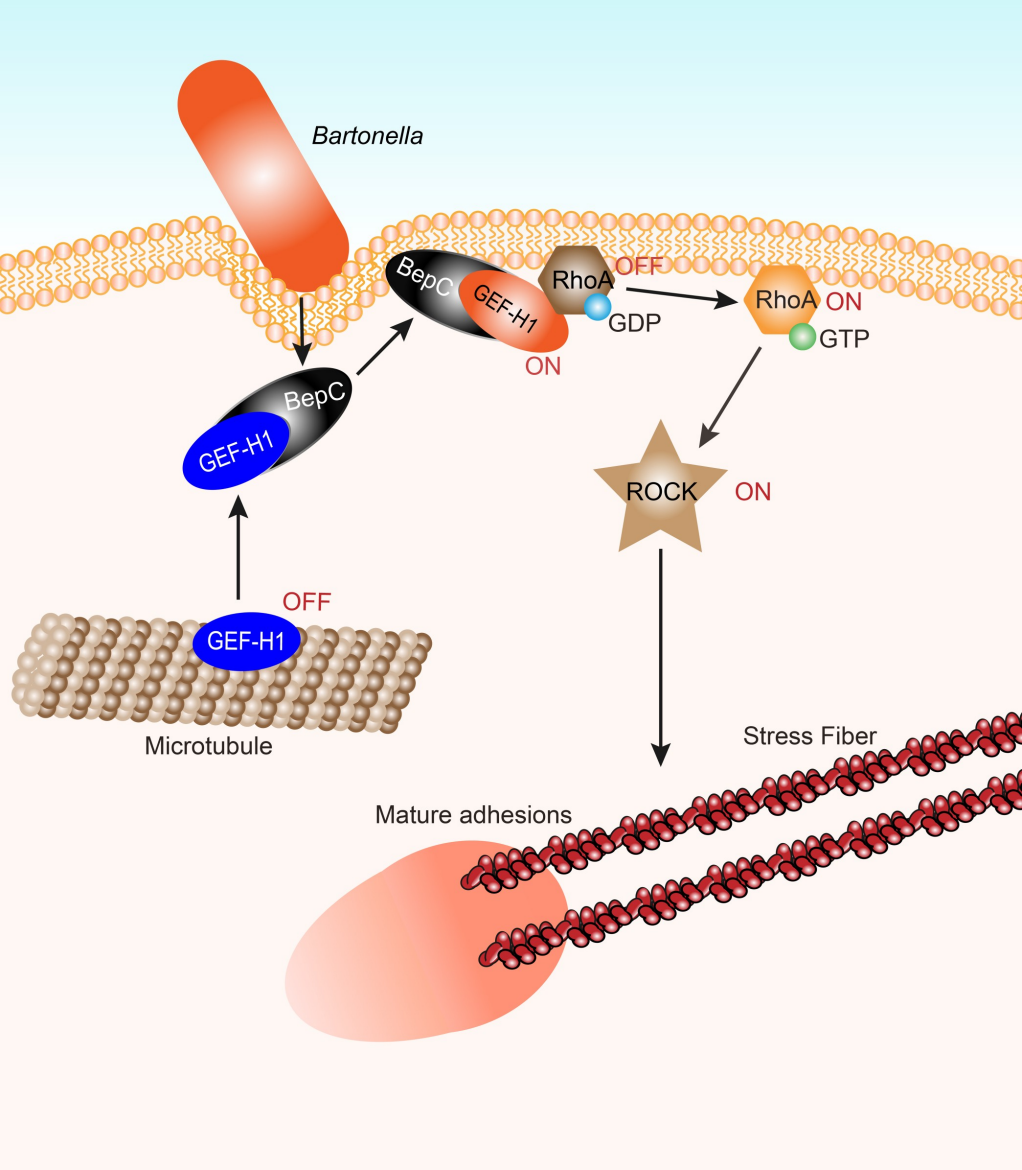
Working model of BepC-induced stress fiber formation. *Bartonella* translocates BepC in host cell by VirB/VirD translocation system. BepC targets GEF-H1 and results its dissociation from microtubule so that GEF-H1 restores its GEF function on RhoA. And then, BID domain of BepC directs BepC/GEF-H1 complex to plasma membrane where GEF-H1 can activate RhoA/ROCK pathway and promotes the formation of stress fibers. Excessive stress fiber formation, however, results the maturation of focal adhesion at the rear edge, and eventually causes host cell fragmentation.

Here, we concluded a working model of how BepC induces stress fiber formation and cell fragmentation. FIC determines the binding of BepC with tubulin-associated GEF-H1 and results its dissociation from microtubule, subsequently, BID domain directs the released GEF-H1 to plasma membrane where GEF-H1 can activate RhoA/ROCK pathway and promotes the formation of stress fibers. Excessive stress fiber formation, however, results the maturation of focal adhesion at the rear edge, which broke the fine balance of cell adhesion and cell contractility that leading to cell fragmentation (Fig 8).

BepC-mediated cell fragmentation is known for its detrimental effect on *Bartonella* dissemination *in vivo*. However, it is unreasonable, in the sense of an evolutionary perspective, for *Bartonella* to retain such an effector that is solely detrimental for its pathogenesis. Given that BepC together with BepF facilitate *Bartonella* invasion into host cells through the invasome structure and BepE inhibits BepC-induced cell fragmentation, biological significance of BepC is achieved through inter-Bep cooperation instead of its own action. Specifically, BepC function is to induce stress fiber formation to facilitate *Bartonella* invasion through an invasome strategy, but stabilization of focal adhesion leading to cell fragmentation is a side effect that must be detoxified by BepE. Moreover, it is not yet clear whether BepE only antagonizes BepC-induced cell fragmentation, or whether there is a synergistic effect of BepC and BepE on *Bartonella* pathogenesis.

## Material and methods

### Bacteria and cell lines

*B*. *quintana* JK31 strain and *B*. *tribocorum* strain (strain CIP 105476 / IBS 506) were maintained in our laboratory. *B*. *henselae* ATCC49882 with *Bep* locus deletion strain and its complemented strains with *Bhe*-BepC, *Bqu*-BepC and empty plasmids were provided by Prof. Christoph Dehio (University of Basel). *Bartonella* strains were grown on Columbia Agar Base (Oxoid) plates with 10% defibrinated sheep blood at 35°C and 5% CO_2_ for 2–3 days. *Escherichia coli* strains DH5α and BL21(DE3) were cultured in Luria-Bertani (LB) broth at 37°C with aeration (220 rpm) or on LB agar at 37°C. HeLa cells (ATCC: CCL-2™) and HEK293T cells (ATCC: CRL-1573™) were cultured in Dulbecco's modified Eagle's medium (DMEM) supplemented with 10% fetal bovine serum (FBS), penicillin (100 units/ml), and streptomycin (100 mg/ml) at 37°C with 5% CO_2_. Primary HUVECs were cultured in complete medium supplemented with 10% FBS. MEF cells were obtained from Professor Qiuhua Huang (Shanghai Jiao Tong University) and cultured in DMEM supplemented with 15% fetal bovine serum (FBS), penicillin (100 units/ml), streptomycin (100 mg/ml), 1% NEAA and 1% glutamine at 37°C under 5% CO_2_.

### DNA constructs

*BepA2*, *BepC*, *BepE*, *BepF1*, *BepF2* and *BepC* truncations of *Bqu* strain, *BepC* of *Bhe* strain, and *BepC* of *Btr* strain were amplified and cloned into pcDNA4.0 TO vector, either with Strep or eGFP tag. FLAG-tagged full-length GEF-H1 and GEF-H1 truncations were amplified from human genome and cloned into pcDNA4.0 TO plasmid. The site-directed mutations of *BepC* were created according to the manufacturer’s protocols (Yeasen, China). For prokaryotic expression, the coding sequences of the Rho-binding domain of Rhotekin (amino acids 7–89) (UniProt, Q8C6B2) were synthesized (Shinegene, Shanghai), and whole length of GEF-H1 was amplified by reverse transcription PCR. And then, genes were constructed into PEGX-6P-1. All plasmids are listed in S2 Table.

### Bacterial infection

The day before infection, HUVEC cells were plated on sterile glass slides in 12 well-plates at 1 × 10^5^ cells/ml and cultured in complete medium without any antibiotics. *Bartonella* were harvested from CBA plates with an inoculating loop and re-suspended in 1 ml M199 medium with 10% FBS. The infection was administered with a multiplicity of infection (MOI) of 300 bacteria per cell and incubated in M199 medium with 10% FBS and 10 μM IPTG for 36 h, and then subjected for immunoblot or immunofluorescent detection.

### Immunofluorescence assay

After transfection with indicated plasmids or bacterial infection, Cells were washed with 1×PBS twice, then fixed with 4% paraformaldehyde (PFA) or cold methanol for 20 min, after that they were permeabilized by 0.2% Triton X-100 for 15 min. Subsequently, cells were immunostained with anti-tubulin mouse antibody (1:200, Abcam, USA), anti-GEF-H1 rabbit antibody (1:150, Abcam, USA), anti-FLAG mouse antibody (1:200, Genscript, China) or anti-Paxillin rabbit antibody (1:200, Abcam, USA) overnight at 4°C. Secondary antibodies for immunolabeling include AlexaFluor 488-labelled anti-mouse IgG antibody (1:250, Yeasen, China), AlexaFluor 594-labelled anti-rabbit IgG antibody (1:250, Yeasen, China). For actin staining, cells were labeled with TRITC-phalloidin (1:200, Yeasen, China). Plasma membrane was probed by using 1,1'-Dioctadecyl-3,3,3',3'-tetramethylindocarbocyanine perchlorate (DiIC18(3), Beyotime, China). Golgi apparatus was probed by Golgi-Tracker Red (Beyotime, China). Mitochondria was visualized by Mito-Tracker Red CMXRos (Beyotime, China). Endoplasmic reticulum was detected by ER-Tracker Red (Beyotime, China). Images were captured using a confocal laser scanning microscope (Leica, Germany) or epi-fluorescence microscope (EVOS FL-2, Life technologies, USA).

### Cell fractionation assay

Cell membrane fraction were analyzed as previously described [55]. *Bqu*-BepC and its truncated versions were transfected in HEK293T cells. After 24 h infection, the cells were collected with PBS and centrifuged at 1200 rpm for 5min, and then resuspended in swelling buffer (10 mM Tris [pH 7.5], 15 mM NaCl, 2 mM MgCl2, and protease inhibitor) for 5 min in ice. Whole cell lysate was prepared by mixture with 2×SDS loading buffer. A final concentration of 8.5% sucrose was added into the remaining samples, and treated with 35 strokes by using a dounce homogenizer. The lysate was centrifuged at 500× g for 10 min to separate cell nucleus. The supernatant was centrifuged at 200,000× g for 1 h at 4°C. Membrane proteins (the pellet) and cytoplasm protein (the supernatant) were collected into 1% Triton X-100 buffer. After that, samples were boiled with 2×SDS loading buffer and then analyzed by immunoblotting.

Cytoskeletal fraction was extracted as described previously [54]. HeLa cells stably expressing eGFP-tagged BepC were induced by adding doxycycline if indicated, and then were collected at indicated time point. After that, washed four times in PBS and lysed in cytoskeleton-stabilizing buffer (10 mM PIPES-NaOH pH 6.8, 250 mM sucrose, 3 mM MgCl_2_, 150 mM KCl, 1 mM EGTA, and 1 mM PMSF) containing 0.15% Triton X-100 for 5 min at 37°C. Cell lysates were then centrifuged at 14,000 g at room temperature for 10 min. The supernatant and the pellet were considered as the cytosolic and cytoskeletal fractions, respectively. Subsequently, protein samples were subjected to SDS-PAGE and immunoblots.

### Immunoprecipitation and immunoblotting

For co-immunoprecipitation of cell lysate, HEK293T cells were transfected with the indicated plasmids using liposomal transfection reagent (Yeasen, China). Cells were lysed by RIPA lysis buffer (50mM Tris [pH7.4], 150mM NaCl, 1% NP-40) supplemented with protease inhibitor (Roche, USA), then centrifuged at 5700 rpm for 20 min at 4°C. The clear cell lysate was incubated with FLAG beads (Genscript, China) or Streptactin beads 4FF (SMART life science, China) overnight at 4°C, then washed at least three times with lysis buffer. For co-immunoprecipitation *in vitro*, GST-tagged GEF-H1 was expressed in BL21 (Rosetta DE3) and was purified by glutathione sepharose 4FF beads (SMART life science, China), while strep-tagged BepC was purified from transfected 293T cells. These two purified proteins were mixed into binding buffer (20mM Tris [pH7.5], 150mM NaCl, 1% Triton X-100), and incubated with Streptactin beads 4FF (SMART life science, China) overnight at 4°C. Proteins bounded with beads were boiled in 1.5×SDS loading buffer, then analyzed by immunoblotting. The protein samples were separated using 4%-20% Bis-Tris gels and transferred onto PVDF membrane. The proteins were subsequently detected with anti-FLAG mouse antibody (1:4000, Genscript, China), anti-Calnexin rabbit antibody (1:1000, Abcam, USA), anti-Strep rabbit antibody (1:2000. Genscript, China), anti-GST rabbit antibody (1:1000, Beyotime, China), anti-GEF-H1 rabbit antibody (1:1000, Abcam, USA), anti-GFP rabbit antibody (1:2000, Genscript, China), anti-tubulin mouse antibody (1:6000, Abcam, USA), anti-RhoA rabbit antibody (1:400, Abcam, USA), anti-Rac1 rabbit antibody (1:1000, Abcam, USA) and anti-Cdc42 rabbit antibody (1:1000, Abcam, USA). The HRP-goat anti rabbit (1:10,000, Yeasen, China) and HRP-goat anti mouse (1:10,000, Yeasen, China) antibody were visualized by BeyoECL Plus (Beyotime, China).

### Cell viability detection

Cell viability analysis was implemented using a CCK-8 kit (Yeasen, China). After 24 h of transfection in HEK293T cells with *Bqu*-BepC tagged with Strep and control plasmid (no gene insertion) in 96-well plates with 100 μl DMEM medium, 10 μl CCK-8 liquid was added to each well, after which culture was continued for 2–3 h. Finally, the absorbance was measured at 450 nm with Tecan infinite 200.

### LDH release assay

Cell cytotoxicity was evaluated by measuring the release of lactate dehydrogenase (Beyotime, China). After 24 h transfection with *Bqu*-BepC tagged with Strep and control plasmid (no gene insertion), the cultured supernatant of HEK293T cells was collected and detected using a commercial LDH kit according to the manufacturer’s protocols (Beyotime, China).

### Annexin-V/PI apoptosis assay

HEK293T cells were transfected with *Bqu*-BepC tagged with Strep and control plasmid (no gene insertion) for 24 h, or treated with 25 μM CCCP (Carbonyl cyanide m-chlorophenyl hydrazine, apoptosis inducer) for 12 h as positive control. Cell samples were trypsinized, collected and centrifuged at 2000 rpm to remove the supernatant. Next, cells were resuspended by adding binding buffer gently. The apoptosis and necrosis were subsequently detected using an FITC Annexin-V apoptosis detection kit according to the manufacturer’s instructions (Yeasen, China). Apoptotic cells stained with FITC Annexin-V and propidium iodide (PI) were analyzed using a BD FACSCalibur flow cytometer.

### *Bqu*-BepC Tet3G-on inducible expression cell line

A doxycycline hyclate inducible binary expression system was developed. We constructed two plasmids: pLVX-CMV-Tet3G-IRES-Blasticidin and pLVX-TRE3Gv-BepC-EGFP-PGK-Puromycin. The two plasmids and lentiviral package mix were co-transfected independently into HEK293T cells, after which the virus was harvested at 48 h and 72 h. The day before infection, Hela cells were seeded in six-well plates and grown until 30%-40% confluence, after which they were infected with two lentiviral particles with a MOI of 10 for 72 h. A stable cell pool was then selected by adding 2.0 μg/ml puromycin and 5.0 μg/ml blasticidin in DMEM for 7–9 days. Finally, a monoclonal cell population was generated by limiting dilution in 96-well plates.

### RNA interference

siRNA sequences of GEF-H1, RhoA, Rac1, and Cdc42 were previously described [26,56–58]. Scramble siRNA was used as non-targeting siRNA control. Stable transgenic Hela cells expressing of *Bqu*-BepC were transfected with HiPiFect transfection reagent (QIAGEN) with two duplexes according to the manufacturer’s instructions. At 24 h after the first transfection, cells were re-transfected with the same siRNA oligos, after which 10 μM doxycycline was added to induce *Bqu*-BepC expression in the following day.

### Drug treatment

Stable transgenic Hela cells expressing of *Bqu*-BepC were induced by doxycycline, and then ROCK inhibitor Y-27632 was added into fresh medium and continued to culture for 18 h. Cells were then collected and analyzed by immunofluorescence staining.

### IP-MS

Transfected BepC tagged with Strep was immunoprecipitated from HEK293T cells after 24 h transfection. Immunoprecipitation sample was resolved by SDS-PAGE, and then subjected to in-gel digestion with trypsin prior to mass spectrometric analysis as previously described [59]. LC-MS/MS analyses were conducted on a nano-LC (EASY-nLC 1200, Thermo Scientific, USA) coupled with an LTQ-Orbitrap mass spectrometer (Orbitrap Velos, Thermo Scientific, USA). Raw data files were processed with Mascot and searched against the human protein database (downloaded from UniProt). Experiments were repeated at least three times.

### RhoGTPase activation assays

Rho activity was analyzed using a Rhotekin binding assay as previously reported [60,61]. The GST-RBD protein was expressed in BL21 (Rosetta DE3) and bounded with glutathione sepharose 4FF beads (SMART life science, China). Hela cells with a *Bqu*-BepC Tet3G-on system were induced to express for 16 h, then lysed in lysis buffer for 30 min, after which cell lysates were harvested by centrifugation at 6000 rpm for 20 min at 4°C. The GST fusion protein binding and immunoprecipitation were performed in lysis buffer (50 mM Tris, pH 8.0, 500 mM NaCl, 1% Triton X-100, 0.25% sodium deoxycholate, 0.1% sodium dodecylsulfate (SDS), 10 mM MgCl_2_, and protease inhibitor). GST-RBD-coupled GSH beads were incubated with cell lysates for 1 h at 4°C with rotation, after which the beads were washed three times with lysis buffer and boiled for 10 min in 1.5×SDS loading buffer, and the Rho activity was assessed by immunoblotting with RhoA antibody.

### Statistical analysis

All statistical analyses were performed with GraphPad prism 7.0 software. Data are shown as bar graph (mean ± SD). Statistical significance was determined by using Student's t test for two unpaired groups, and one-way ANOVA with multiple comparisons test for three or more groups (compared with blank data in immunofluorescent data and compared with empty vector in immunoblot data). *p* values less than 0.05 were considered to be statistically significant. Information of number of biological and technical replicates can be found in the figure legends when appropriate.

## Supporting information

S1 Fig *Bqu*-BepC causes non-apoptotic and necrotic cytotoxicity. (A) Expression of BepC impaired cell viability detected by CCK-8. (B) LDH release assay showed no significant difference of lytic cell death caused by BepC expression. (C) Annexin V/PI co-staining confirmed BepC-induced cytotoxicity was not apoptosis. Blank indicated non transfected cells and empty vector was plasmid without gene insertion. Expression of BepC was detected by immunoblots. CCCP was apoptosis inducer. One-way ANOVA with multiple comparisons test was used. “**” p < 0.001. All assays were performed more than three times independently, and representative data are shown. Values shown are means ± SD.
(TIF)

S2 Fig *Bqu*-BepC-induced cell fragmentation is not cell type specific. HUVEC, HEK293T, and mouse MEF cells transfected with BepC were stained with TRITC-phalloidin. All tested cell types developed stress fiber formation and cell fragmentation. Assays were performed more than three times. Data from one representative experiment data (n = 3) were shown. Bar = 10 μm.
(TIF)

S3 Fig Orthologous BepC shows general effects on stress fiber formation. (A) Transfection of BepC from Bhe and Btr in Hela cells caused stress fiber formation, but a less potent effect of Btr BepC on cell fragmentation was observed. (B) Percentage of cell fragmentation with the orthologous BepC was analyzed (cells in ten randomly selected visual fields were calculated). All assays were performed more than three times. Data from one representative experiment data (n = 3) were shown. Values shown are means ± SD. Bar = 10 μm.
(TIF)

S4 Fig BepC binds with GEF-H1 *in vitro*. Strep tagged BepC was purified from transfected 293T cells. GEF-H1 fused with GST tag was expressed in *E*.*coli* and purified by using glutathione sepharose beads. Prokaryotic expression of GEF-H1 was verified by immunoblot using a GEF-H1 antibody and GST tag antibody. Co-immunoprecipitation *in vitro* was performed by using Streptactin beads. The unbounded Streptactin beads and GST tag protein served as negative controls.
(TIF)

S1 Movie Cell fragmentation induced by ectopic expression of *Bqu*-BepC. The eGFP-BepC was transfected in Hela cells. The white arrow showed cells with defect in detachment of rear edge that leading trailing tails and cell fragmentation. Daughter cell bodies separated from parent cells were indicated by yellow arrow. The yellow circle indicated that BepC resulted in decreased cell rigidity and cell nucleus bulging. Representative of 3 independent experiments.
(MP4)

S1 Table Protein list identified by LC-MS/MS. The list of identified proteins by LC-MS/MS in the immunoprecipitated samples of BepC and SseK2. Spectral counts of those detected proteins from three biological replicates were reported and processed to get protein ratios and p-values.
(XLSX)

S2 Table Plasmids used in this work. (DOCX)
