# Supplementary figures and images for: Bartonella type IV secretion effector BepC induces stress fiber formation through activation of GEF-H1
Source: PLoS Pathog. 2021 Jan 28;17(1):e1009065. doi: 10.1371/journal.ppat.1009065 (PMC7842913; doi:10.1371/journal.ppat.1009065)

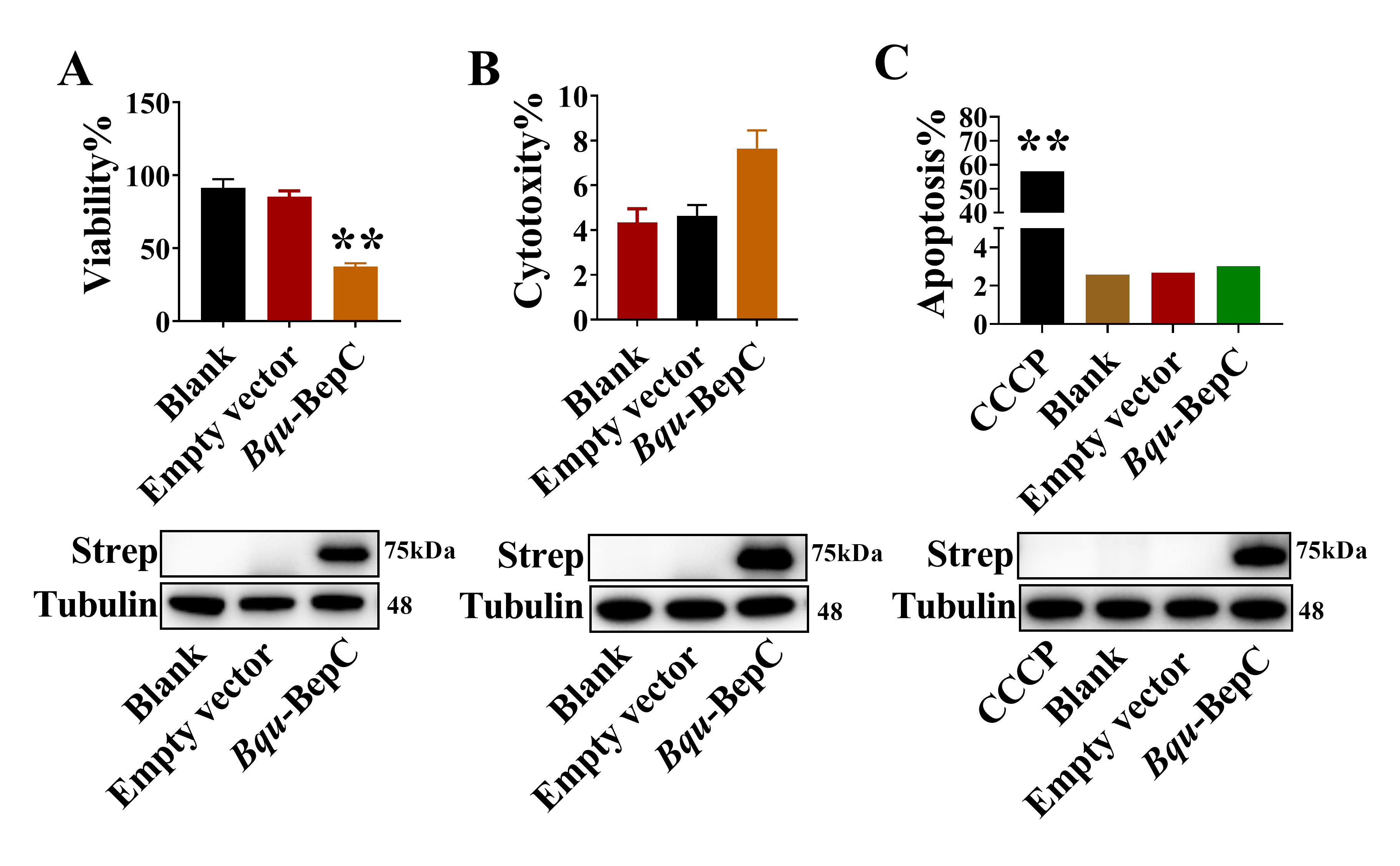

Supplement: S1 Fig — (A) Expression of BepC impaired cell viability detected by CCK-8. (B) LDH release assay showed no significant difference of lytic cell death caused by BepC expression. (C) Annexin V/PI co-staining confirmed BepC-induced cytotoxicity was not apoptosis. Blank indicated non transfected cells and empty vector was plasmid without gene insertion. Expression of BepC was detected by immunoblots. CCCP was apoptosis inducer. One-way ANOVA with multiple comparisons test was used. “**” p < 0.001. All assays were performed more than three times independently, and representative data are shown. Values shown are means ± SD. (TIF) [file ppat.1009065.s001.tif]

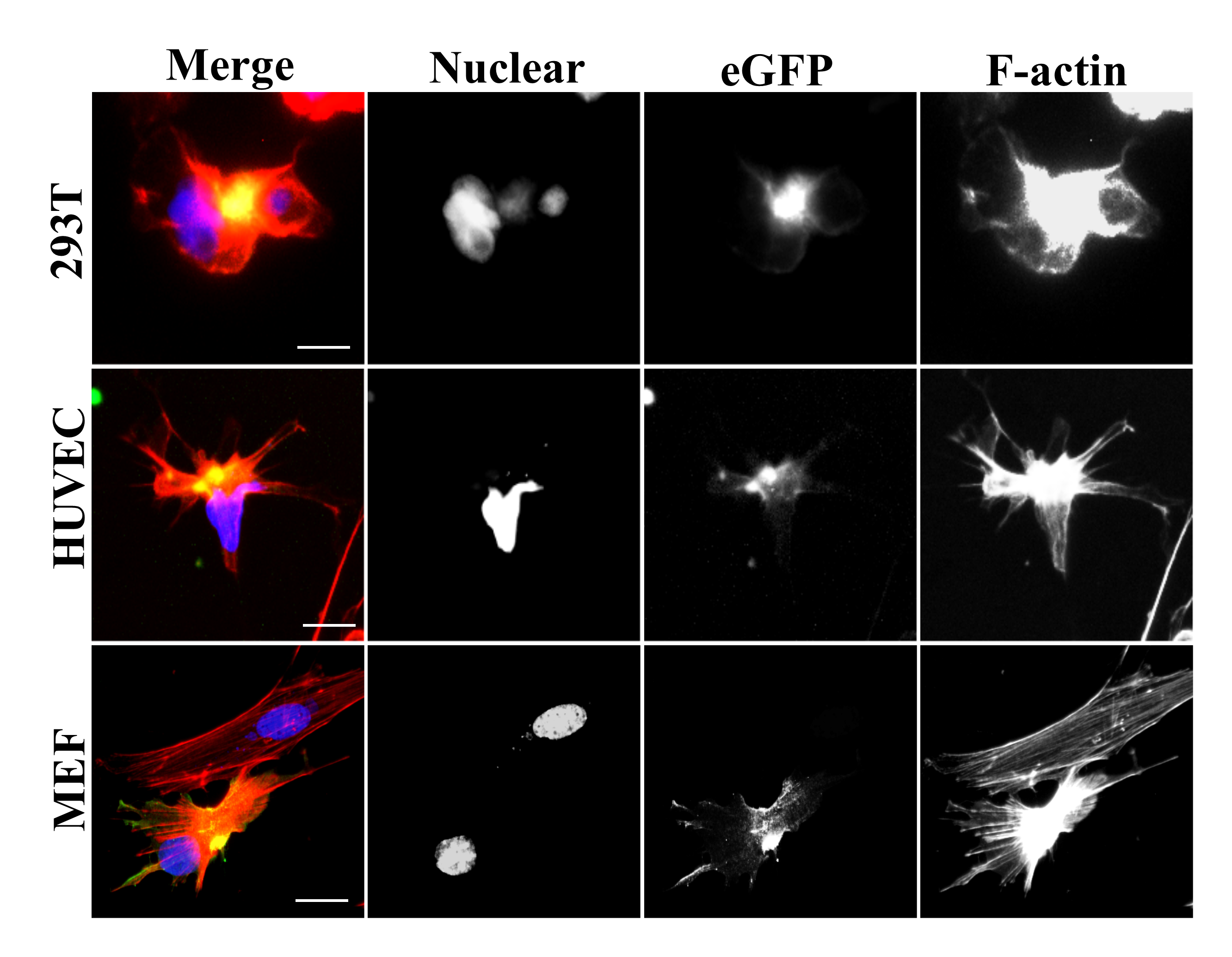

Supplement: S2 Fig — HUVEC, HEK293T, and mouse MEF cells transfected with BepC were stained with TRITC-phalloidin. All tested cell types developed stress fiber formation and cell fragmentation. Assays were performed more than three times. Data from one representative experiment data (n = 3) were shown. Bar = 10 μm. (TIF) [file ppat.1009065.s002.tif]

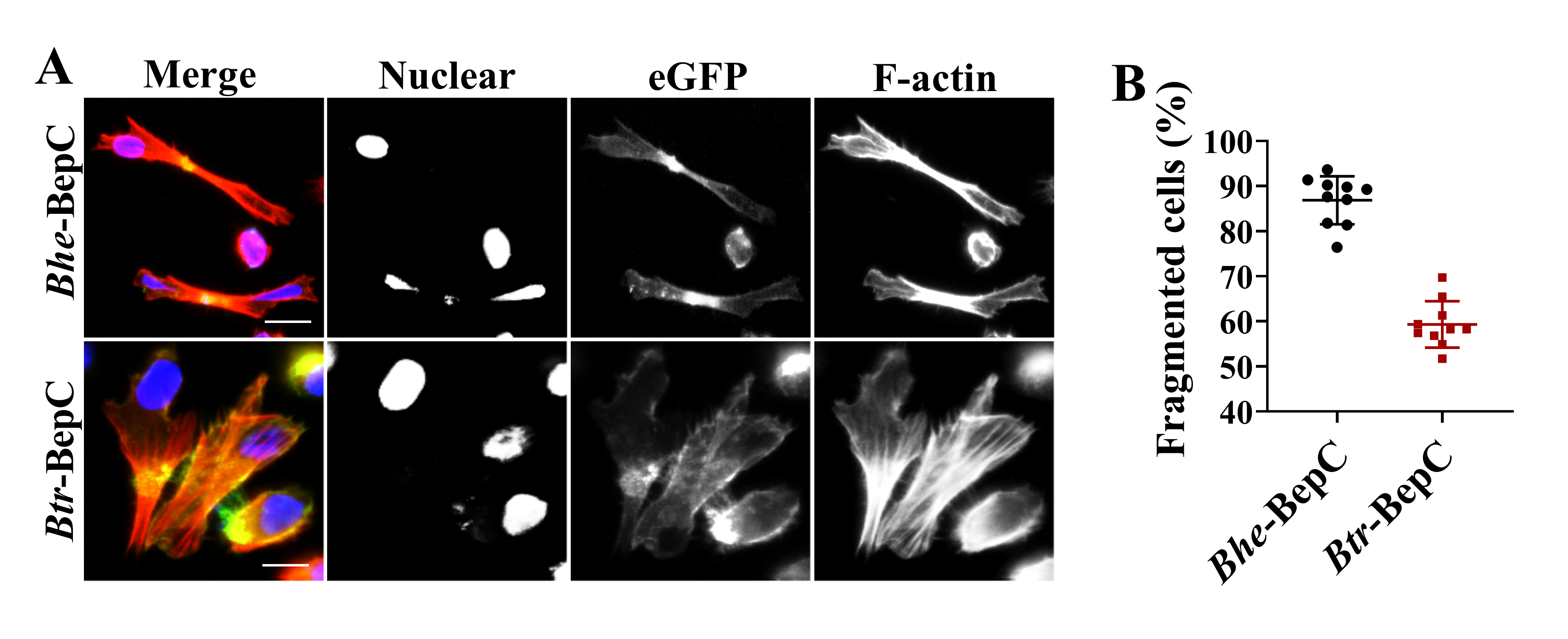

Supplement: S3 Fig — (A) Transfection of BepC from Bhe and Btr in Hela cells caused stress fiber formation, but a less potent effect of Btr BepC on cell fragmentation was observed. (B) Percentage of cell fragmentation with the orthologous BepC was analyzed (cells in ten randomly selected visual fields were calculated). All assays were performed more than three times. Data from one representative experiment data (n = 3) were shown. Values shown are means ± SD. Bar = 10 μm. (TIF) [file ppat.1009065.s003.tif]

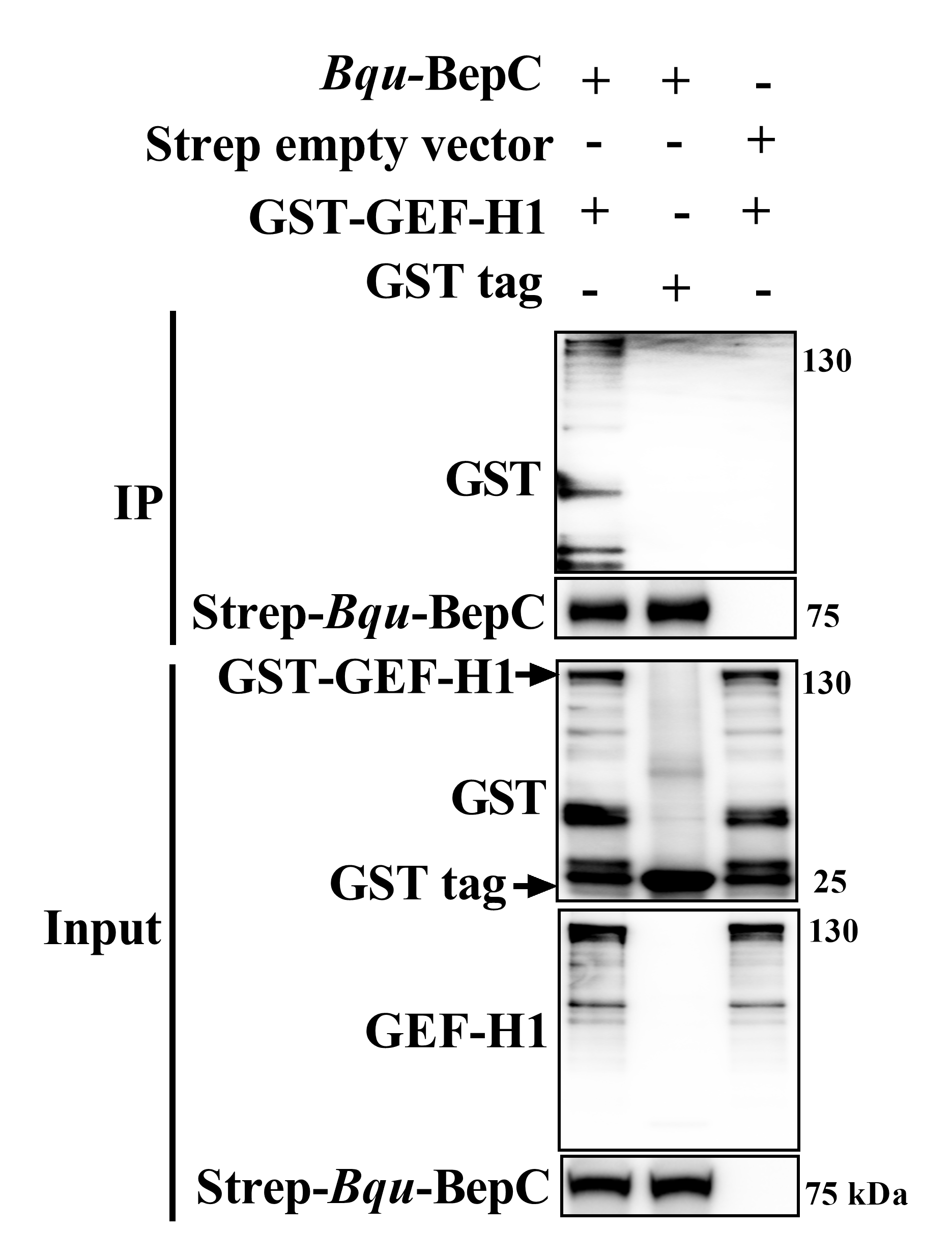

Supplement: S4 Fig — Strep tagged BepC was purified from transfected 293T cells. GEF-H1 fused with GST tag was expressed in E.coli and purified by using glutathione sepharose beads. Prokaryotic expression of GEF-H1 was verified by immunoblot using a GEF-H1 antibody and GST tag antibody. Co-immunoprecipitation in vitro was performed by using Streptactin beads. The unbounded Streptactin beads and GST tag protein served as negative controls. (TIF) [file ppat.1009065.s004.tif]
