# Supplementary material for: Bartonella type IV secretion effector BepC induces stress fiber formation through activation of GEF-H1
Source: PLoS Pathog. 2021 Jan 28;17(1):e1009065. doi: 10.1371/journal.ppat.1009065 (PMC7842913; doi:10.1371/journal.ppat.1009065)
Supplement: S2 Table — (DOCX) [file ppat.1009065.s007.docx]

**S1 Table. Plasmids using in this work**

| **Plasmid** | **Description** | **Source** |
| --- | --- | --- |
| pcDNA4.0-eGFP/Strep/FLAG vector | Mammalian expression vector with eGFP, Strep or FLAG tag | This study |
| pcDNA4.0-eGFP-*Bqu*-BepC | Mammalian expression vector for BepC with eGFP tag from *Bartonella quintana* | This study |
| pcDNA4.0-eGFP-*Bqu*-BepE | Mammalian expression vector for BepE with eGFP tag from *Bartonella quintana* | This study |
| pcDNA4.0-eGFP-*Bqu*-BepA2 | Mammalian expression vector for BepA2 with eGFP tag from *Bartonella quintana* | This study |
| pcDNA4.0-eGFP-*Bqu*-BepF1 | Mammalian expression vector for BepF1 with eGFP tag from *Bartonella quintana* | This study |
| pcDNA4.0-eGFP-*Bqu*-BepF2 | Mammalian expression vector for BepF2 with eGFP tag from *Bartonella quintana* | This study |
| pcDNA4.0-eGFP-*Bhe*-BepC | Mammalian expression vector for BepE with eGFP tag from *Bartonella henselae* | This study |
| pcDNA4.0-eGFP-*Btr*-BepC | Mammalian expression vector for BepE with eGFP tag from *Bartonella tribocorum* | This study |
| pSIM091-FLAG-*Bhe*-BepC | Prokaryotic expression vector for BepC with FLAG tag from *Bartonella henselae* | Gifted from Pro. Christoph Dehio |
| pSIM054-FLAG-*Bqu*-BepC | Prokaryotic expression vector for BepC with FLAG tag from *Bartonella quintana* | Gifted from Pro. Christoph Dehio |
| pBZ485_empty vector | Prokaryotic expression vector for negative control | Gifted from Pro. Christoph Dehio |
| pcDNA4.0-eGFP-*Bqu*-BepC-FIC | Mammalian expression vector for BepC-FIC (1-300aa) with eGFP tag from *Bartonella quintana* | This study |
| pcDNA4.0-eGFP-*Bqu*-BepC-BID | Mammalian expression vector for BepC-BID (301-533aa) with eGFP tag from *Bartonella quintana* | This study |
| pcDNA4.0-eGFP-*Bqu*-BepC-N152S | Mammalian expression vector for BepC-N152S with eGFP tag from *Bartonella quintana* | This study |
| pcDNA4.0-eGFP-*Bqu*-BepC-P147A | Mammalian expression vector for BepC-P147A with eGFP tag from *Bartonella quintana* | This study |
| pcDNA4.0-eGFP-*Bqu*-BepC-H164R | Mammalian expression vector for BepC-H164R with eGFP tag from *Bartonella quintana* | This study |
| pcDNA4.0-eGFP-*Bqu*-BepC-G151S | Mammalian expression vector for BepC-G151S with eGFP tag from *Bartonella quintana* | This study |
| pcDNA4.0-eGFP-*Bqu*-BepC-F148I | Mammalian expression vector for BepC-F148I with eGFP tag from *Bartonella quintana* | This study |
| pcDNA4.0-eGFP-*Bqu*-BepC-ΔFIC | Mammalian expression vector for BepC-ΔFIC with eGFP tag from *Bartonella quintana* | This study |
| pcDNA4.0-Strep-*Bqu*-BepC | Mammalian expression vector for BepC with Strep tag from *Bartonella quintana* | This study |
| pcDNA4.0-Strep-*Bqu*-BepC-FIC | Mammalian expression vector for BepC-FIC (1-300aa) with Strep tag from *Bartonella quintana* | This study |
| pcDNA4.0-Strep-*Bqu*-BepC-BID | Mammalian expression vector for BepC-BID (301-533aa) with Strep tag from *Bartonella quintana* | This study |
| pcDNA4.0-Strep-*Bqu*-BepC-N152S | Mammalian expression vector for BepC-N152S with Strep tag from *Bartonella quintana* | This study |
| pcDNA4.0-Strep-*Bqu*-BepC-P147A | Mammalian expression vector for BepC-P147A with Strep tag from *Bartonella quintana* | This study |
| pcDNA4.0-Strep-*Bqu*-BepC-H164R | Mammalian expression vector for BepC-H164R with Strep tag from *Bartonella quintana* | This study |
| pcDNA4.0-Strep-*Bqu*-BepC-G151S | Mammalian expression vector for BepC-G151S with Strep tag from *Bartonella quintana* | This study |
| pcDNA4.0-Strep-*Bqu*-BepC-F148I | Mammalian expression vector for BepC-F148I with Strep tag from *Bartonella quintana* | This study |
| pcDNA4.0-Strep-*Bqu*-BepC-ΔFIC | Mammalian expression vector for BepC-ΔFIC with Strep tag from *Bartonella quintana* | This study |
| pcDNA4.0-FLAG-GEF-H1 | Mammalian expression vector for GEF-H1 with FLAG tag from HUVEC cell | This study |
| PGEX6P-1-GEF-H1 | Prokaryotic expression vector for GEF-H1 with GST tag from HUVEC cell | This study |
| pcDNA4.0-FLAG-GEF-H1(1-234) | Mammalian expression vector for GEF-H1 (1-234aa) with FLAG tag from HUVEC cell | This study |
| pcDNA4.0-FLAG-GEF-H1(235-572) | Mammalian expression vector for GEF-H1 (235-572aa) with FLAG tag from HUVEC cell | This study |
| pcDNA4.0-FLAG-GEF-H1(1-572) | Mammalian expression vector for GEF-H1 (1-572aa) with FLAG tag from HUVEC cell | This study |
| pcDNA4.0-FLAG-GEF-H1(235-986) | Mammalian expression vector for GEF-H1 (235-986aa) with FLAG tag from HUVEC cell | This study |
| pcDNA4.0-FLAG-GEF-H1(573-986) | Mammalian expression vector for GEF-H1 (573-986aa) with FLAG tag from HUVEC cell | This study |
| PGEX-6P-RBD | Prokaryotic expression vector for RBD (7-89aa) with GST tag from Mouse Rhotekin | This study |
| pLVX-CMV-Tet3G-IRES-Blasticidin | Lentiviral expression vector for inducer plasmid | This study |
| pLVX-TRE3Gv-BepC-eGFP-PGK-Puromycin | Lentiviral expression vector for *Bqu*-BepC with eGFP tag | This study |
